# Supplementary material for: Mixed Solvents in Multilayer Ceramic Capacitors (MLCC) Electronic Paste and Their Effects on the Properties of Organic Vehicle
Source: Polymers (Basel). 2022 Feb 11;14(4):685. doi: 10.3390/polym14040685 (PMC8879018; doi:10.3390/polym14040685)
Supplement: Supplementary file 1 [file polymers-14-00685-s001.zip › supplementary/supporting.pdf]

Supporting materials

Mixed Solvents in Multilayer Ceramic Capacitors (MLCC) Electronic Paste and Their Effects on the Properties of Organic Vehicle

Ruolong Gan [1], Junrong Li [1,*], Xiuhua Cao [2], Jun Huang [2] and Liying Qian [1,*]

1. School of Light Industry and Engineering, South China University of Technology, Guangzhou 510640, China; 202021028911@mail.scut.edu.cn
2. State Key Laboratory of Advanced Materials and Electronic Components, Zhaoqing 526020, China; caoxh@china-fenghua.com (X.C.); squallhj1234@163.com (J.H.)

* Correspondence: lljrr@scut.edu.cn (J.L.); lyqian@scut.edu.cn (L.Q.); Tel.: +86-20-87111770 (J. L.)

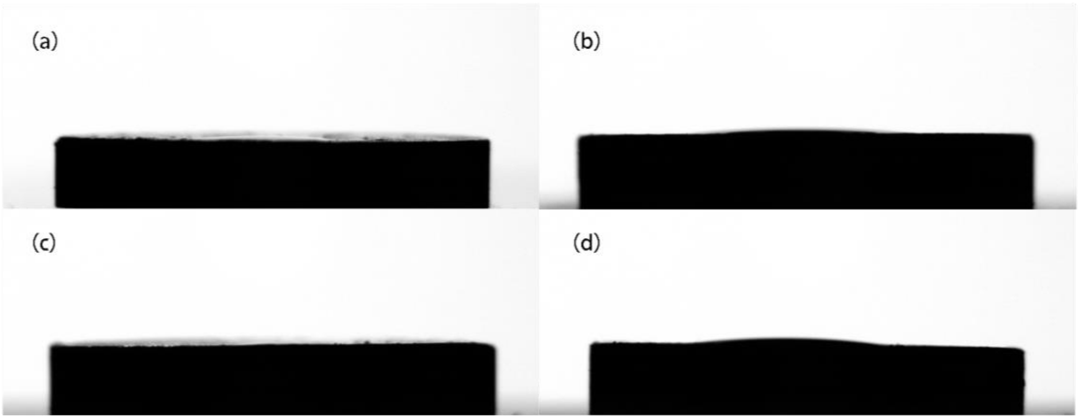

Figure S1. Contact angles of the mixed solvents on glass powder sheet (a) T-DMF; (b)T-DBAC; (c) T-CAC; (d) T-DGBE

According to the Figure S1, the wettability of the four mixed solvents to the glass powder sheet is complete wetting. It shows that the mixed solvent has commendable wettability to the glass powder sheet, the mixed use of the two solvents will not affect the performance of the electronic paste, and ensure the normal sintering process of the electronic paste.

Video S1-S4 show the fast wetting process.

Table S1 Three-dimensional solubility parameters of solvents and monomers of resins

Sample	δ_d （$J^{1/2}$·$cm^{-3/2}$）	δ_p （$J^{1/2}$·$cm^{-3/2}$）	δ_h （$J^{1/2}$·$cm^{-3/2}$）	δ（$J^{1/2}$·$cm^{-3/2}$）
MMA	16.54	5.20	8.62	19.36
BMA	16.62	3.44	7.01	18.36
T	17.12	5.50	11.35	21.26

DGBE	15.96	6.95	10.64	20.40
DBAC	15.96	4.09	8.18	18.40
CAC	15.96	4.71	10.64	19.75
DMF	17.39	13.71	11.25	24.84
